# Genome Sequences of *Gordonia* Phages BaxterFox, Kita, Nymphadora, and Yeezy

**DOI:** 10.1128/genomeA.00600-16

**Published:** 2016-08-11

**Authors:** Welkin H. Pope, Sharanya Bandla, Alexandra K. Colbert, Fiona G. Eichinger, Michelle B. Gamburg, Stavroula G. Horiates, Jerrica M. Jamison, Dana R. Julian, Whitney A. Moore, Pranav Murthy, Meghan C. Powell, Sydney V. Smith, Nadia Mezghani, Katherine A. Milliken, Paige K. Thompson, Chelsea L. Toner, Megan C. Ulbrich, Emily C. Furbee, Sarah R. Grubb, Marcie H. Warner, Matthew T. Montgomery, Rebecca A. Garlena, Daniel A. Russell, Deborah Jacobs-Sera, Graham F. Hatfull

**Affiliations:** Department of Biological Sciences, University of Pittsburgh, Pittsburgh, Pennsylvania, USA

## Abstract

*Gordonia* phages BaxterFox, Kita, Nymphadora, and Yeezy are newly characterized phages of *Gordonia terrae*, isolated from soil samples in Pittsburgh, Pennsylvania. These phages have genome lengths between 50,346 and 53,717 bp, and encode on average 84 predicted proteins. All have G+C content of 66.6%.

## GENOME ANNOUNCEMENT

The *Actinobacteria* is a large and diverse bacterial phylum that includes many species of both medical and environmental interest. Phages have been isolated for many species within the group but relatively few genome sequences are available, other than for *Mycobacterium smegmatis* for which hundreds of phage genomes sequences have been reported (1). *Gordonia terrae* is a reasonably close relative of the mycobacteria (both are in the order *Corynebacteria*) present in soil and environmental samples, and is being used by students in the Science Education Alliance-Phage Hunters Advancing Genomics and Evolutionary Science (SEA-PHAGES) program to isolate and genomically characterize phages of *G. terrae* 3612 (1, 2).

Four bacteriophages—BaxterFox, Yeezy, Nymphadora, and Kita—were isolated from soil samples from Pittsburgh, PA using *G. terrae* 3612 as a host, either by direct plating (BaxterFox, Yeezy) or by enrichment (Nymphadora, Kita). Following plaque purification, amplification, and dsDNA extraction, each phage genome was sequenced using an Illumina MiSeq and 140 bp single-end reads were assembled using Newbler. Reads were assembled into a single major contig for each phage, to generate genomes of 53,717 bp, 50,346 bp, 53,431 bp, and 51,884 bp with coverages of 259-fold, 827-fold, 650-fold, and 966-fold, for BaxterFox, Kita, Nymphadora, and Yeezy, respectively; all have similar G+C% contents (66.5% to 66.7%). All four genomes have defined ends, and Kita and Nymphadora have 10-base 3′ single strand extensions (5′-CGGCTGGGGA), whereas BaxterFox and Yeezy have 11-base 3′ single strand extensions (5′-TGCCAGGGGGA). Protein-coding genes were predicted using Glimmer (3) and GeneMark (4) and putative functions were assigned using BLASTp, HHpred (5), and Phamerator (6).

The four genomes share substantial nucleotide sequence similarity with each other, which in pairwise BLASTn alignments span from 44% (Yeezy and Nymphadora) to 75% (Kita and Nymphadora); they are not closely related to previously described phages or prophages, although lysis and DNA methylase genes are closely related to those in a putative *Gordonia* sp. KTR9 prophage (7). In general, the tail genes are closely related in all four genomes, but terminase large and small subunits, portal, protease, and major capsid subunit genes in BaxterFox and Yeezy are unrelated to those in Kita and Nymphadora. Interestingly, these differences influence the virion morphologies, and although all have long flexible noncontractile tails, BaxterFox and Yeezy have isometric heads, whereas Kita and Nymphadora have prolate heads. Furthermore, the major capsid subunit proteins of Kita and Nymphadora share ~65% amino acid identity with the major capsid subunits of cluster I mycobacteriophages, which have similarly prolate heads. The differences in the terminases presumably also give rise to the different genome ends.

Baxterfox and Yeezy code for similar tyrosine integrases and are predicted to integrate into an *attB* site overlapping a tRNA^ala^ gene (KTR9_RS07590) that is occupied by a putative prophage in *Gordonia* sp. KTR9 (7). The tyrosine integrases encoded by Kita and Nymphadora are distantly relatedly to the BaxterFox/Yeezy integrases, and Kita and Nymphadora are predicted to integrate into an *attB* site overlapping a tRNA^ser^ gene (KTR9_RS02590 in *Gordonia* sp. KTR9).

### Accession number(s).

The BaxterFox, Nymphadora, Kita, and Yeezy genomes are available from GenBank under the accession numbers KU963263, KU963255, KU963257, and KU963249, respectively.
